# 122. Optimization of Antibiotic Time-Outs Within a Health System

**DOI:** 10.1093/ofid/ofab466.324

**Published:** 2021-12-04

**Authors:** Ashley Long, Sara Revolinski, Anne R Daniels

**Affiliations:** 1 Memorial Hermann Southeast Hospital, Houston, Texas; 2 Medical College of Wisconsin, Milwaukee, WI; 3 Froedtert and Medical College of Wisconsin, Milwaukee, Wisconsin

## Abstract

**Background:**

The Infectious Diseases Society of America estimates that up to 50% of antibiotic use in hospitals is inappropriate. In order to assist with reducing inappropriate antibiotic use, the Centers for Disease Control and Prevention has recommended systemic evaluation of ongoing antibiotic therapy need, such as antibiotic time-outs (ATOs), be implemented. This has further been supported by the Joint Commission in their antimicrobial stewardship medication management standard. Our system implemented a prescriber-led ATO process in 2018, but documented completion of the ATO remained low. Due to this, pharmacists were integrated into the ATO process with the goal of increasing completion rates.

**Methods:**

This pre-post interventional study analyzed the impact of an antibiotic time out process implemented for patients receiving piperacillin/tazobactam (P/T) or cefepime (CEF) for a minimum of 48 hours. The pre-group (Jan-April 2018) had ATOs completed by the primary medical team, while pharmacists completed the ATO in the post group (Jan-April 2020). For each group, a computerized alert prompted completion of the ATO in the electronic health record (EHR). The alert included systematic questions to assess the need for continued P/T and CEF use. The primary outcome was percentage of ATO documentation completed. Secondary outcomes included inappropriate continuation of P/T and CEF and de-escalation within 24 hours after ATO completion.

**Results:**

A total of 248 and 234 patients in the pre- and post-groups were included, respectively. Significantly more ATOs were documented in the post-group compared to the pre-group (65.5% vs 48.5%, p< 0.001). Similarly, inappropriate continuation of P/T and CEF after the ATO process was significantly lower in the post-group compared to the pre-group (11.6% vs 64.0%, p< 0.001). While not statistically significant, there was a trend toward increased de-escalation in the post-group within 24 hours of ATO completion (58.9% vs 47.9%, p=0.105).

**Conclusion:**

A pharmacist-led ATO process reduced inappropriate use of P/T and CEF compared to a prescriber-led process. Incorporating pharmacists into an ATO process may optimize antimicrobial stewardship outcomes.

**Disclosures:**

**All Authors**: No reported disclosures

